# Supplementary material for: A Thermal-Stable Protein Nanoparticle That Stimulates Long Lasting Humoral Immune Response
Source: Vaccines (Basel). 2023 Feb 13;11(2):426. doi: 10.3390/vaccines11020426 (PMC9962852; doi:10.3390/vaccines11020426)
Supplement: Supplementary file 1 [file vaccines-11-00426-s001.zip › vaccines-2169839-supplementary-update-2.23.pdf]

vaccines

Supplementary material

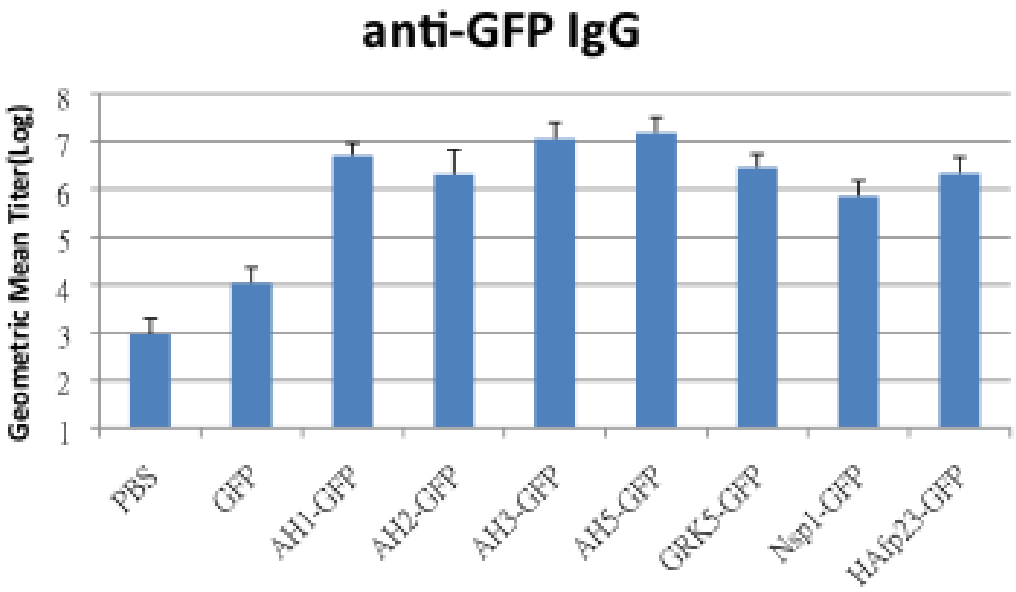

Figure S1. Anti-GFP antibody titers of mice immunized by fusion proteins composed of amphipathic helical peptide and GFP. Mice were immunized twice with 14 days apart by intramuscular injection of 20 μg of fusion protein indicated. Sera collected at day 14 to day 16 post booster immunization were used for ELISA analysis. Data were compiled from 3 different individual experiments under the same experimental procedure. The ELISA plates were coated with 10μg/ml GFP and the 2nd antibody was used at a concentration of 1:2000 dilution. The other procedures were executed as standard protocol. (N=4 or 5) The result suggested the fusion of amphipathic helical peptide onto GFP increases the antigenicity of GFP by 2~3 log.

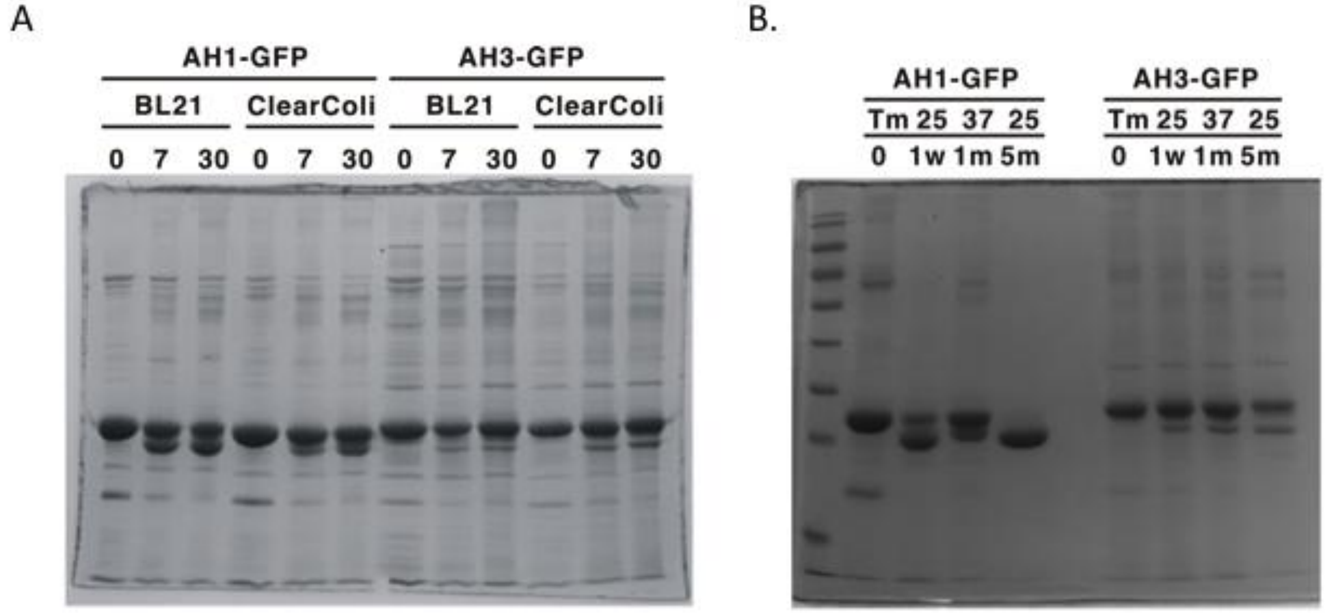

Figure S2. Examining the protein stability of AH1-GFP and AH3-GFP fusion protein in ambient temperature. AH1-GFP and AH3-GFP transformed into BL21(DE3) or ClearColi competent cells for protein expression were induced at 15°C overnight and then following the standard protocol for protein purification. Proteins were dialyzed into 1xPBS in a dialysis membrane with MWCO of 3000 Da overnight. (A) Purified proteins were kept in 37°C for one week or one month and then analyzed by SDS-PAGE and Coomassie blue staining. (B) Fusion proteins from ClearColi were kept in 37°C one month or RT (25°C) for one week or 5 months and then analyzed by SDS-PAGE and Coomassie blue staining. Results from figure S2A indicates AH1-GFP fusion protein stability in 37°C is reduced in the presence of LPS, but not AH3-GFP. Also, from Figure S2B, the result indicates the AH3-GFP is stable in both 25 °C and 37°C up to 5 months. But for AH1-GFP fusion protein, more than 70% of the protein was degraded into GFP in a week and completely converted to GFP in 5 months in 25 °C. The results suggest AH3-GFP is stable in both ambient temperatures tested.

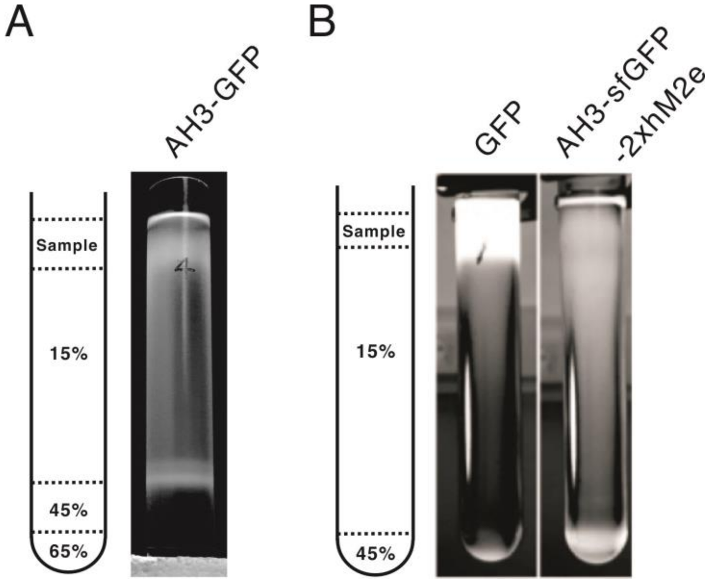

Figure S3. The hydrophobicity of the AH3-GFP protein nanoparticle was analyzed using density gradient ultracentrifugation. Protein samples were loaded on top of sucrose step gradients and

then centrifuged at 35K rpm for 2 hrs in a SW41-Ti rotor. After centrifugation, fluorescent protein distribution was detected under 450nm LED light and pictures taken for analysis by ImageJ from NIH. (A) The centrifuge tube was first loaded with 1ml 65% sucrose (w/v), 2 ml 45% sucrose (w/v) and 7 ml 15% sucrose (w/v) consecutively and then topped with bacterial lysate containing AH3-GFP fusion protein for analysis. (B) The centrifuge tube was loaded first with 1ml 45% (w/v) sucrose solution and then followed with 9ml of 15% (w/v) sucrose solution. One milliliter soluble fraction from each sample was loaded on top of the centrifuge tube before ultracentrifugation as described. Results of two samples were shown, GFP and AH3-sfGFP-2xhM2e. The results showed the GFP protein alone remained in the top of the centrifuge tube, but AH3-sfGFP-2xhM2e had been sedimented to the junction between 15%/45% sucrose solutions.

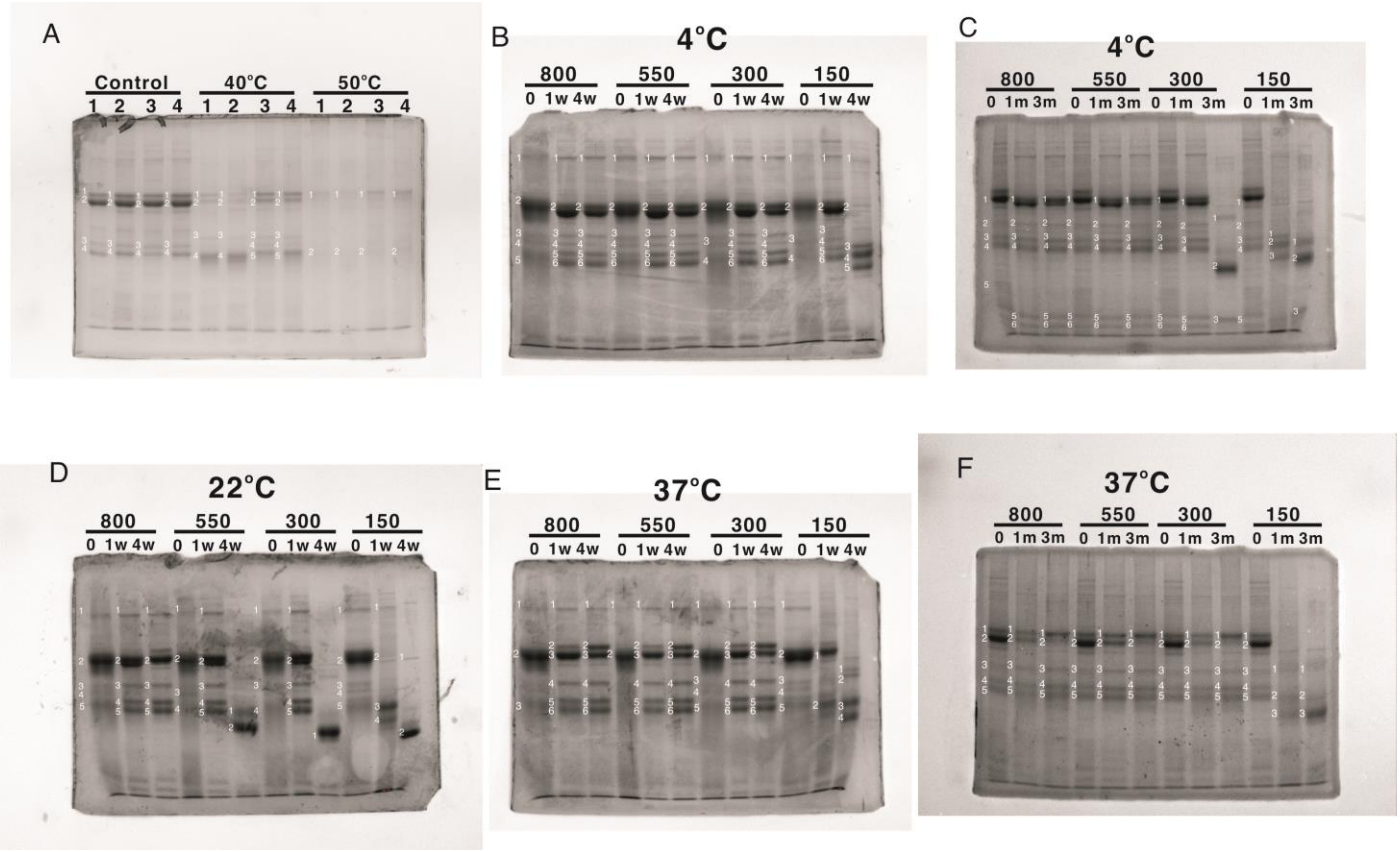

Figure S4. Densitometry quantification of protein bands from Figure 7. The protein bands from each lane were numbered in white. The bands were quantified using Plot lanes function of ImageJ. The relative percentge of each bands in the same lane being quantified was summarized in Table S1. The figures A, B, C, D, E, and F were the labeled, uncropped counter parts of Figure 7A, 7B, 7C, 7D, 7E, and 7F.

Table S1. The relative percentages of protein bands being quantifed from SDS-PAGE gels A-F of Figure S4.

S4A		Control				40°C				50°C			
	Band #	1	2	3	4	1	2	3	4	1	2	3	4
	1	17.2	21.4	18.5	20.7	9.5	4.7	24.6	21.5	63.5	57.7	76.0	68.7
	2	56.4	54.3	60.2	55.9	10.3	6.8	29.4	25.1	36.5	42.3	24.0	31.3
	3	7.5	7.2	8.3	6.1	4.0	2.2	3.5	3.0				
	4	18.9	17.1	12.9	17.3	76.2	86.4	4.0	3.4				
	5							38.5	46.9				

S4B	4 °C	800 mM			550 mM			300 mM			150 mM		
	Band #	0	1w	4w	0	1w	4w	0	1w	4w	0	1w	4w
	1	4.1	6.5	6.5	6.6	10.9	7.3	9.5	7.1	5.7	6.1	6.4	2.9
	2	81.1	66.2	62.5	69.7	59.1	64.7	76.3	65.4	62.7	83.4	66.0	2.1
	3	2.0	2.9	3.0	2.7	2.5	3.3	2.8	3.1	4.3	3.3	1.8	17.9
	4	1.7	1.7	0.9	1.8	1.8	1.2	11.4	1.4	1.2	7.3	5.1	40.7
	5	11.0	7.1	8.4	4.4	8.5	8.0		7.9	9.3		14.7	36.4
	6		15.7	18.7	14.7	17.1	15.5		15.0	16.8		5.9	

S4C	4 °C	800 mM			550 mM			300 mM			150 mM		
	Band #	0	1m	3m	0	1m	3m	0	1m	3m	0	1m	3m
	1	70.7	67.7	64.2	68.5	66.0	58.1	69.6	68.2	8.1	71.3	15.3	27.3
	2	2.8	2.7	2.7	3.5	3.9	2.6	3.0	3.2	80.6	3.0	48.4	58.6
	3	6.3	6.6	8.8	6.9	6.7	9.7	7.3	7.4	11.3	5.6	36.3	14.0
	4	14.1	14.7	15.2	16.7	15.4	20.9	13.9	13.8		14.6		
	5	6.1	5.1	5.2	2.9	4.7	6.1	3.8	4.3		5.5		
	6		3.1	3.8	1.5	3.2	2.5	2.3	3.0				

S4D	22 °C	800 mM			550 mM			300 mM			150 mM		
	Band #	0	1w	4w	0	1w	4w	0	1w	4w	0	1w	4w
	1	3.6	7.2	5.6	4.3	6.2	37.3	3.4	6.2	100.0	3.8	3.8	5.8
	2	61.2	64.5	48.9	78.6	48.7	62.7	71.0	57.1		60.7	1.5	94.2
	3	2.3	3.7	5.9	2.4	5.2		8.5	3.6		2.5	50.0	
	4	3.8	8.6	13.5	14.7	11.2		17.0	10.9		3.8	44.8	
	5	29.1	16.0	26.2		28.7			22.2		29.2		

S4E	37 °C	800 mM			550 mM			300 mM			150 mM		
	Band #	0	1w	4w	0	1w	4w	0	1w	4w	0	1w	4w
	1	5.5	5.1	3.1	2.3	6.9	9.8	3.1	4.7	6.5	5.6	54.9	6.1
	2	82.9	12.7	21.3	97.7	9.1	20.9	82.2	10.6	18.0	79.8	45.1	9.4
	3	11.6	40.4	31.7		41.8	30.7	1.3	41.8	29.6	2.6		40.0
	4		5.8	6.8		6.4	7.2	1.2	7.6	6.2	2.2		44.5
	5		11.9	12.7		11.1	11.1	12.1	12.9	12.0	9.7		
	6		24.2	24.3		24.8	20.2		22.4	27.8			

S4F	37 °C	800 mM			550 mM			300 mM			150 mM		
	Band #	0	1m	3m	0	1m	3m	0	1m	3m	0	1m	3m
	1	18.4	24.5	33.8	17.4	22.3	33.9	20.7	22.5	36.7	17.2	3.6	2.9
	2	55.1	30.4	16.2	54.0	30.5	13.4	56.1	30.3	11.9	55.1	40.7	18.6
	3	3.4	7.3	6.8	4.2	6.5	6.7	3.1	6.0	5.3	3.9	55.7	78.5
	4	7.5	12.9	15.9	7.8	14.2	15.5	6.0	13.0	16.0	7.7		
	5	15.6	25.0	27.4	16.5	26.5	30.4	14.1	28.2	30.1	16.0		
